# Altered Functional Connectivity Density in Subtypes of Parkinson’s Disease

**DOI:** 10.3389/fnhum.2017.00458

**Published:** 2017-09-20

**Authors:** Xiaofei Hu, Yuchao Jiang, Xiaomei Jiang, Jiuquan Zhang, Minglong Liang, Jing Li, Yanling Zhang, Dezhong Yao, Cheng Luo, Jian Wang

**Affiliations:** ^1^Department of Radiology, Southwest Hospital, Third Military Medical University Chongqing, China; ^2^Key Laboratory for NeuroInformation of Ministry of Education, School of Life Science and Technology, University of Electronic Science and Technology of China Chengdu, China; ^3^Department of Centre for Disease Prevention and Control, Chengdu Military Region Chengdu, China; ^4^Department of Neurology, Southwest Hospital, Third Military Medical University Chongqing, China

**Keywords:** Parkinson’s disease, subtypes, resting state, fMRI, functional connectivity density

## Abstract

Parkinson’s disease (PD) can be classified into tremor-dominant and akinetic-rigid subtypes, each of which exhibits a unique clinical course and prognosis. The neural basis for these disparate manifestations is not well-understood, however. This study comprehensively investigated the altered functional connectivity patterns of these two subtypes. Twenty-five tremor-dominant patients, 25 akinetic-rigid patients and 26 normal control subjects participated in this study. Resting-state functional MRI data were analyzed using functional connectivity density (FCD) and seed-based functional connectivity approaches. Correlations between neuroimaging measures and clinical variables were also calculated. Compared with normal control, increased global FCD occurred most extensively in frontal lobe and cerebellum in both subtypes. Compared with akinetic-rigid patients, the tremor-dominant patients showed significantly increased global FCD in the cerebellum and decreased global FCD in portions of the bilateral frontal lobe. Furthermore, different subtypes demonstrated different cerebello-cortical functional connectivity patterns. Moreover, the identified FCD and functional connectivity correlated significantly with clinical variables in the PD patients, and particularly the FCD indices distinguished the different subtypes with high sensitivity (95%) and specificity (80%). These findings indicate that the functional connectivity patterns in the cerebellum and frontal lobe are altered in both subtypes of PD, especially cerebellum are highly related to tremor.

## Introduction

Parkinson’s disease (PD) is a neurodegenerative disorder that is typically characterized by motor signs that include tremor, akinesia, and rigidity (Lees et al., 2009). It can be classified as the akinetic-rigid (AR) or tremor-dominant (TD) subtype based on the predominant motor signs (Zaidel et al., 2009). This symptomatic heterogeneity of the different PD subtypes may have unique pathophysiological substrates. Uncovering the neural substrates that account for this heterogeneity is critically important for advancing our knowledge of PD and to identify effective therapies. However, the brain mechanisms that underlie the specific PD subtypes are not well-established.

Previous functional neuroimaging studies have demonstrated that the neurobiological substrates of parkinsonian tremor differ from those of akinesia and rigidity. Previous studies have documented structural and/or functional changes in the two PD subtypes. A voxel-based morphometry study showed that TD patients had reduced gray matter volume in the cerebellum (Herman et al., 2013). Using R-fMRI, our research team compared TD and akinetic/rigid-predominant subtypes of PD and found distinct regional homogeneity patterns of spontaneous brain activity in the striato-thalamo-cortical loop and the cerebello-thalamo-cortical loop between these two subtypes of PD (Zhang et al., 2015c). However, these approaches have examined connectivity within a single network [e.g., the default mode network (DMN)] (Tan et al., 2015) or in local regions (e.g., the ReHo) (Zhang et al., 2015c) and have not considered alterations in whole-brain networks. Therefore, in the present study, we sought to fully delineate the abnormal functional connectivity (FC) networks of the two subtypes by searching the entire brain connectome at a refined voxel level. Functional connectivity density (FCD) is a recently developed, data-driven (i.e., with no need for *a priori* hypotheses) method for identifying the number of functional connections of each brain voxel (Tomasi and Volkow, 2010, 2012; Luo et al., 2014). High FCD values for particular voxels indicate that those voxels are functionally connected to a greater number of other brain voxels and play more important roles (Hub) than others in information processing (Tomasi and Volkow, 2010, 2011; Chen et al., 2015). Based on the neighboring relationships between brain voxels, FCD can be further divided into local and global FCD (Tomasi and Volkow, 2010). Local FCD of a voxel reflects local functional integration, and global FCD reflects functional integration across the whole brain. Data-driven global FCD is useful for exploratory studies and that it can guide seed-voxel correlation analyses when we do not have clear hypotheses for the seed regions (Tomasi and Volkow, 2010, 2012). Although the FCD analysis allows us to identify brain sites that exhibit abnormal FC in two subtypes, it does not provide insight into the locations to which these abnormal connections are linked. To trace these locations, seed-based FC analyses were performed.

In the current study, combined FCD and seed-based FC analyses were performed to fully characterize the abnormal brain networks in the two subtypes. Finally, we correlated the network changes with clinical characteristics of the patients and used receiver operating characteristic (ROC) analysis to evaluate the ability of the FCD measures to discriminate the PD subtypes. In a previously published study, we observed differences in interhemispheric coordination between PD subtypes (Zhang et al., 2015b). Based on prior studies (Lewis et al., 2011; Zhang et al., 2015b) we hypothesized that the functional integration in the cerebellum and motor-related cortical regions would be altered in the two subtypes.

## Materials and Methods

### Subjects

The present study was approved by the Medical Research Ethics Committee of the Third Military Medical University (Chongqing, China), and written informed consent was obtained from all subjects. Fifty right-handed PD patients [25 AR patients (M:9/F:16, Age(y): 65.12 ± 10.11)] and 25 TD patients [M:9/F:17, Age(y): 60.28 ± 11.14] were recruited from the movement disorders outpatient clinic of Southwest Hospital. Each PD subject was diagnosed by an experienced movement disorder specialist according to the United Kingdom Parkinson’s Disease Society Brain Bank criteria (Hughes et al., 1992). Importantly, all PD patients were screened to exclude patients with visual hallucination symptoms.

In the current study, only those patients who could tolerate long periods of immobility in the MRI scanner were included. Subsequently, motor performance was quantitatively and accurately assessed in detail by using the motor section (part III) of the Unified Parkinson’s Disease Rating Scale (UPDRS) (Fahn et al., 1987) and used to classify PD subjects by subtype. Specifically, the PD subjects were classified as either TD or AR based on the ratio of the mean tremor score (TD score) to the mean akinetic-rigidity score (AR score) (Eggers et al., 2011; Lewis et al., 2011). Just like the previously published study (Zhang et al., 2015b), for each patient, we computed a mean tremor score for 9 tremor items (right and left arm tremor from patient history, and lips or chin tremor, tremor in all 4 limbs, and activity in both arms or postural tremor, on examination) and a mean score for 5 AR items (falling, freezing, and walking difficulty, from patient history, and gait and postural instability, on exam-ination). Briefly, patients were classified as having TD-PD when the ratio of the mean tremor score to the mean AR score was ≥1.5 and as having AR-PD when this ratio was ≤1 (Eggers et al., 2011; Lewis et al., 2011). In addition, a Mini Mental State Examination (MMSE) score > 28 and a Montreal Cognitive Assessment (MoCA) score > 26 were required for all subjects to ensure that no patients met the criteria for dementia (Dubois et al., 2007). The patient exclusion criteria included secondary parkinsonism, atypical parkinsonian disease, and pre-existing neurological or psychiatric disorders (including seizures, aphasia, neglect, substantial sensory disturbances, severe depression, or claustrophobia). Meanwhile, patients with severe tremor which might disturb the MRI procedure were excluded.

All patients were substituted with dopaminergic treatment but none were treated with antidepressants. Three of 25 TD patients were also receiving low-dose anticholinergics (benzhexol, 0.5 mg/3 times daily).

In addition, 26 healthy controls (HCs) [M:9/F:17, Age(y): 59.84 ± 7.01] were recruited from among local individuals who volunteered to participate in scientific studies. All of the control subjects had normal neurological examination results and underwent neuropsychological testing prior to the MRI scan. The exclusion criteria were the same as those applied to the patients.

### Data Acquisition

Functional images were acquired using a 3.0 T Siemens Tim Trio whole-body MRI system (Siemens Medical Solutions, Erlangen, Germany). Subjects were instructed to stay awake and close their eyes, and to try not to think of anything. All subjects confirmed they did not fall asleep during the MRI procedures. Foam padding and earplugs were used to reduce head motion and scanner noise. Imaging data were collected transversely by using an echo-planar imaging (EPI) sequence with the following settings: TR = 2000 ms, TE = 30 ms, flip angle = 90°, FOV = 192 mm × 192 mm, in-plane matrix = 64 × 64, thickness = 3 mm, voxel size = 3.0 mm × 3.0 mm × 3.0 mm. For each subject, a total of 240 volumes were acquired with scan time of 480 s. To minimize the impact of dopaminergic medication, the MRI scans were performed during a relatively hypodopaminergic state (12 h after a last dose of dopaminergic treatment). Other medications (anticholinergics) were withdrawn for 12 h prior to scanning (ALI–MELKKILÄ et al., 1993; Khor and Hsu, 2007).

### Data Preprocessing

Data preprocessing was carried out using Neuroscience Information Toolbox (NIT^1^). A series of conventional preprocessing steps were performed, which include (1) discarding first five volumes; (2) slice-timing correction; (3) head-motion correction; (4) normalization with an EPI template in the Montreal Neurological Institute (MNI) space; (5) linear detrending; (6) temporal filtering (band-pass 0.01–0.08 Hz); and (7) Regressing out six motion parameters, white matter and cerebrospinal fluid. Participants were excluded from further analyses if head motion exceeded 2.0 mm or 2.0°. We further evaluated the mean absolute displacement of each volume as compared with the previous volume (Zhang et al., 2015b). The average/standard deviation of frame-wise displacement (FD) for each group is present at Supplementary Table 1. All the three groups showed very slight head motion (mean FD < 0.1). In addition, there is no differences among the three groups (*F* = 1.302, *p* = 0.278, ANOVA). Considering that the censoring may lead to fewer degrees of freedom and introduce noisy (Power et al., 2015), we did not apply the volume censoring in the preprocessing (Supplementary Data).

### Functional Connectivity Density Analysis

**FIGURE 1:**
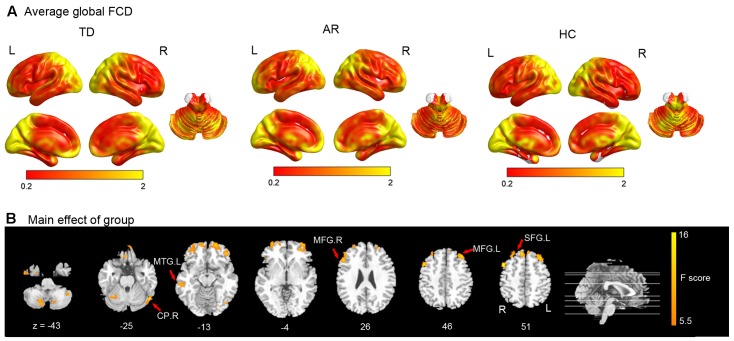
Results of the FCD analyses. **(A)** Shows the spatial distribution of the average global functional connectivity density (FCD) for TD, AR, and HC. **(B)** Show result of the significant main effect of group on FCD (one-way ANCOVA, *p* < 0.005 corrected for multiple comparison at the cluster level, AlphSim corrected). CP, cerebellum posterior lobe; MTG, middle temporal gyrus; MFG, middle frontal gyrus; SFG, superior frontal gyrus.

According to the approach introduced by Tomasi and Volkow (2010) we used custom-written software in Neuroscience Information Toolbox (NIT^1^) to evaluate the individual voxel-wise FCD maps. The global FCD of a given voxel was defined as the number of functional connections between that voxel and all other voxels. The local FCD was obtained using a “growing” algorithm. In this algorithm, given a voxel χ0, an additional voxel χj was added to the list of neighbors of χ0 if it was adjacent to a voxel that was linked to χ0 by a continuous path of functionally connected voxels and the correlation coefficient between χ0 and χj was >0.6. This calculation was repeated for all voxels that were adjacent to the neighbors of χ0 in an iterative manner until no new neighbors could be added to the list. The local FCD (intraregional) of χ0 was defined as the number of elements in the list of neighbors. Therefore, the global FCD reflected the whole brain FC, whereas the local FCD reflected local FC. The threshold of the correlation coefficient is a key parameter in FCD analysis. Based on prior knowledge, the threshold was set at 0.6 (Supplementary Data and Figure 1; Tomasi and Volkow, 2010, 2011). For each subject, a global FCD map and a local FCD map were computed. For standardization purposes, individual FCD maps were divided by the mean FCD value. Prior to the group-level analyses, we applied 6-mm FWHM spatial smoothing to the normalized FCD maps.

### Seed-Based Functional Connectivity Analysis

The brain sites identified by FCD group comparisons between the two subtypes were used as seeds in subsequent FC analyses. Specifically, a sphere ROI was generated for each of the brain sites, the center of which corresponded to the peak voxel (radius = 3 mm); the mean BOLD time series was then extracted and correlated to the time series of all of the voxels in the brain (Li et al., 2015). In this way, FC maps of each seed were produced; these maps were transformed by Fisher’s r-to-z transformation to improve normality.

### Statistical Analysis

One-way analyses of variance and Chi-squared tests were used to analyze demographic characteristics among the three groups, and two-sample *t*-tests were used to perform *post hoc* analysis. All tests were two-tailed, and *p* < 0.05 was considered statistically significant. These analyses were performed with SPSS software (version 20.0; SPSS, Chicago, IL, United States).

To determine the effect of group, a voxel-based one-way analysis of covariance (ANCOVA) was used with age, gender, and education level as covariates in both the FCD and FC analyses using REST software. The mean FCD and FC values were extracted for *post hoc* analyses using the least significant difference (LSD) *t*-test. Multiple comparisons correction was performed using a height threshold (*p* < 0.005) of individual voxel and a cluster size based on Monte Carlo simulations (Ledberg et al., 1998), which corresponds to cluster-level *p* < 0.05 by AlphaSim correction. The AFNI AlphaSim program^2^ was used to correct for multiple comparisons.

We evaluated the clinical data for normality and then performed Spearman rank correlation analyses to investigate the relationships between each of the clinical variables (duration, age of onset, UPDRS-III, H-Y scale score, TD score, and AR score) and the altered functional brain properties separately (the normalized FCD values of the ROIs and the average z-score of the altered FC). ROC curves were computed using the mean global FCD differences between the TD patients and the AR patients. The discriminatory performance of each index was evaluated separately. Additionally, the indices were combined using a binary logistic regression model, and the performance of this model was also assessed. In each case, the area under the ROC curve (AUC) and the 95% confidence interval (CI) were calculated using non-parametric methods. Spearman rank correlation analyses and ROC analysis were performed with SPSS software.

## Results

### Demographic and Clinical Data of Participants

There were no significant differences among the three groups in terms of gender, age, or educational level and no significant differences between the TD and AR patients in terms of the duration of disease, Hoehn and Yahr scale score or UPDRS-III score (**Table 1**).

**Table 1:** Demographic information and clinical characteristics of study subjects.

Variable (Mean ± SD)	TD (*n* = 25)	AR (*n* = 25)	HC (*n* = 26)	*P*-value
Gender (male/female)	12/13	9/16	9/17	0.56^a^
Age (year)	60.28 ± 11.14	65.12 ± 10.11	59.84 ± 7.01	0.10^b^
Education (year)	8.16 ± 2.62	9.08 ± 3.68	9.57 ± 2.51	0.23^b^
MMSE	29.00 ± 0.95	29.23 ± 0.91	28.92 ± 0.70	0.41^b^
MoCA	26.44 ± 1.29.	26.40 ± 1.15	26.96 ± 1.24	0.19^b^
Age of onset	55.20 ± 13.03	58.40 ± 11.29	–	0.36^c^
Disease duration (year)	5.26 ± 5.01	6.66 ± 5.08	–	0.33^c^
Hoehn and Yahr Scale	2.14 ± 0.71	2.46 ± 0.49	–	0.07^c^
UPDRS-III score	33.56 ± 15.88	35.84 ± 13.96	–	0.59^c^
Tremor score	2.17 ± 0.68	1.08 ± 0.22	–	<0.011^c^
AR score	1.56 ± 0.46	2.01 ± 0.41	–	0.001^c^
Levodopa dosage (mg/day)	305.80 ± 98.55	341.40 ± 71.52	–	0.15^c^

*MMSE, Mini Mental State Examination; ^a^The *p*-value for gender distribution in the three groups was calculated using chi-squared test. ^b^The *p*-value was calculated using one-way analysis of variance tests. ^c^The *p*-value was calculate using two-tail two-sample *t*-test. TD, tremor-dominant Parkinson’s disease; AR, akinetic-rigidity Parkinson’s disease; HC, healthy control*.

One-way analysis of variance did not reveal any significant differences in head motion when measured by either the mean or maximum relative displacement [*F*(2,69), translational mean: *F* = 0.039, *p* = 0.961; translational max: *F* = 0.493, *p* = 0.613; rotational mean: *F* = 2.93, *p* = 0.061; rotational max: *F* = 1.012, *p* = 0.369]. In addition, there is no differences among three groups in term of FD [*F* = 1.302, *p* = 0.278, ANOVA, *F*(2,69)], indicating that the PD patients and controls exhibited similar head motion characteristics.

### Functional Connectivity Density Analysis

Using the averaged local and global FCD maps, we found that those regions with high local and global FCD (Hubs) were mainly distributed bilaterally, with maximal magnitude in the posterior cingulate, occipital and prefrontal cortices (**Figure 1A** and **Table 2**).

**Table 2:** The difference of global FCD among TDs, ARs, and HCs.

Region	MNI coordinates			Peak *F*-value	Peak *T*-value	Cluster size (mm^3^)
	*x*	*y*	*z*			
**Main effect of group**						
L_cerebellum posterior lobe	-45	-72	-21	13.17		1377
	-18	-66	-54	8.8		864
R_cerebellum inferior lobule	12	-75	-48	9.53		1026
R_middle temporal gyrus	60	-18	-18	9.12		918
R/L_superior frontal gyrus	9	39	51	14.57		999
	-18	57	-18	10.4		1296
	-42	54	-9	11.5		3510
	30	63	-9	9.8		1647
	-6	37	51	12		810
R_middle frontal gyrus	51	15	48	16.09		1269
	51	24	24	10.5		2322
	30	33	48	8.91		999
L_middle frontal gyrus	-33	27	54	12.78		2565
**TDs > ARs**						
L_cerebellum anterior lobe	-24	-48	-18		3.66	1242
R_superior frontal gyrus	24	39	48		-4.26	1593
R_middle frontal gyrus	48	39	30		-3.5	1593
L_inferior frontal gyrus	-54	24	30		-4.23	1107
**TDs > HCs**						
R_cerebellum posterior lobe	24	-69	-51		4.1	2160
	33	-78	-57		3.96	621
	36	-54	-24		3.64	1323
L_superior frontal gyrus	-3	48	-33		3.81	1593
R_fusiform gyrus	39	-27	-21		3.71	1161
**ARs > HCs**						
L_cerebellum posterior lobe	-48	-63	-27		-4.28	1836
R_middle temporal gyrus	63	-30	-12		4.76	1593
	54	-3	-33		3.79	729
L/R_frontal lobe	-42	51	-12		4.41	8208
	-12	69	18		3.42	918
	6	33	-30		3.81	2052
	3	54	-18		3.7	1323
R_inferior frontal gyrus	51	24	24		4.16	1863
	42	30	-12		4.25	837
R_middle frontal gyrus	33	30	54		3.34	2565
R_middle frontal gyrus	51	12	48		5.22	1809
L_middle frontal gyrus	-36	36	42		4.5	2916

*The results are the main effect of group on global functional connectivity density [one-way ANCOVA, *p* < 0.005 corrected for multiple comparison at the cluster level, AlphSim corrected, *F*(2,69)] and the comparison of global functional connectivity density between TDs and ARs (*post hoc* two-sample *t*-test, *p* < 0.005 corrected for multiple comparison at the cluster level, AlphSim corrected)*.

For comparison among three groups of global FCD, the brain regions that showed a significant main effect (one-way ANCOVA, *p* < 0.005, AlphaSim corrected) predominantly included prefrontal regions (superior/middle frontal gyrus), the temporal cortex (middle temporal gyrus), and the cerebellum (Inferior and posterior lobe) (**Figure 1B** and **Table 2**).

**FIGURE 2:**
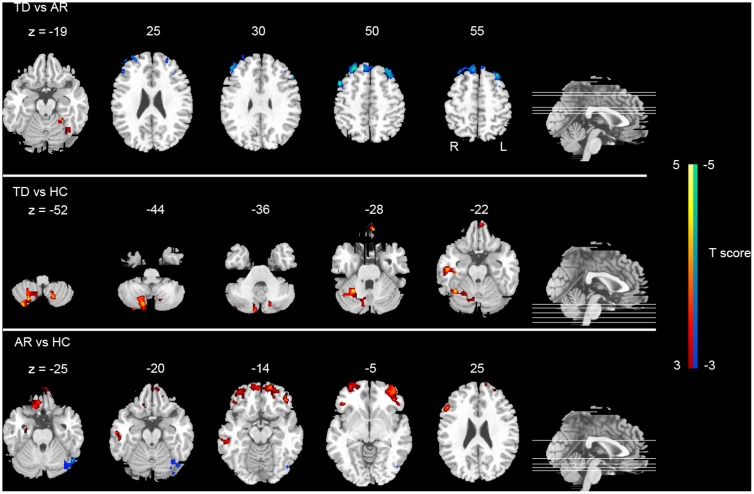
Statistical group differences (*post hoc* analyses) of FCD. The statistical differences (*t* score) of *post hoc* analysis (*post hoc* two-sample *t*-test, *p* < 0.005 corrected for multiple comparison at the cluster level, AlphSim corrected). Color bar represents the *t*-value.

*Post hoc* analyses were applied to identify significant pairwise differences between three pairs of groups (*post hoc* two-sample *t*-test, *p* < 0.005, AlphaSim corrected). (1) TD vs. AR. The TD patients had significantly decreased global FCD in the left inferior frontal gyrus, right middle frontal gyrus and right superior frontal gyrus, and increased global FCD in the cerebellum anterior lobe relative to the AR patients (**Figure 2** and **Table 2**). (2) TD vs. HC. Compared with HCs, the TD patients had significantly increased global FCD in the right cerebellum posterior lobe, left superior frontal gyrus and right fusiform gyrus (**Figure 2** and **Table 2**). (3) AR vs. HC. Compared with the HCs, the AR patients exhibited significantly increased global FCD in the left posterior lobe of the cerebellum, the right middle temporal gyrus, the bilateral middle frontal gyrus, and the right inferior frontal gyrus (**Figure 2** and **Table 2**).

No significant differences in local FCD were found among groups. Thus, the four regions that exhibited significant differences in global FCD between TD and AR (the left cerebellum anterior lobe, right superior frontal gyrus, right middle frontal gyrus, and left inferior frontal gyrus) were defined as seeds in the following FC analysis.

**FIGURE 3:**
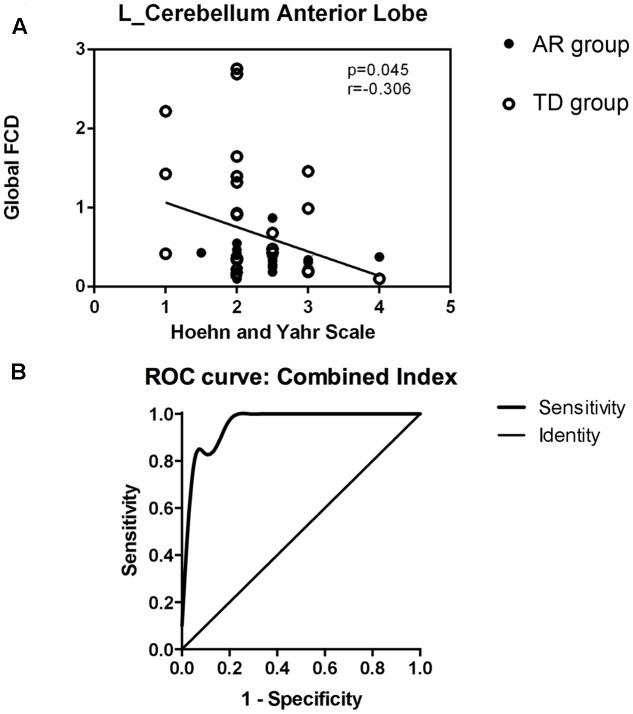
**(A)** Show the correlations between regions with altered global FCD and clinical data in PD group (TDs and ARs) (spearman rank correlation analyses, *p* < 0.05). **(B)** Shows receiver operating characteristic (ROC) curve of the combination of the four FCD indexes for distinguishing PD subtypes patients.

We correlated the clinical variables with the normalized FCD values of the four regions just mentioned (the left cerebellum anterior lobe, right superior frontal gyrus, right middle frontal gyrus, and left inferior frontal gyrus) for the TD and AR patients separately. In the PD group (TD and AR patients), a significant negative correlation was identified between H-Y scale score and the FCD value of the cerebellum anterior lobe (**Figure 3A**).

### Discriminatory Performance of the Functional Connectivity Density

The results of the ROC analysis indicated that the discriminatory performance of each FCD index was far lower than the inclusion of the four FCD indices (**Table 3**) and the inclusion of the four FCD indices in a binary logistic regression model had the highest power to discriminate the AR patients from the TD patients, with an AUC of 0.96 (95% CI = 0.91–1.00; **Figure 3B** and **Table 3**). At a cutoff of 0.23, the sensitivity and specificity were 95.2 and 80.9%, respectively (**Table 3**).

**Table 3:** Discriminatory performance of the functional connectivity density.

FCD index	Area under ROC curve	Significance level *p*-value	Cutoff value	Sensitivity (%)	Specificity (%)
Cerebellum anterior lobe	0.73 (0.56, 0.90)	0.01	0.47	61.9 (38.4, 81.9)	90.4 (69.6, 98.8)
Superior frontal gyrus	0.83 (0.79, 0.96)	<0.001	0.51	95.2 (76.1, 99.8)	52.4 (29.7, 74,3)
Middle frontal gyrus	0.85 (0.74, 0.96)	<0.0001	0.64	66.6 (43.0, 85.4)	90.5 (69.6, 98.8)
Inferior frontal gyrus	0.85 (0.73, 0.96)	0.0001	0.38	90.4 (69.6, 98.8)	66.7 (43.0, 85.4)
Combination of the four indexes	0.96 (0.91, 1.00)	<0.0001	0.23	95.2 (76.2, 99.8)	80.9 (58.1, 94.5)

*Numbers in parentheses are 95% confidence intervals*.

### Functional Connectivity Analysis of Regions of Different FCD

**FIGURE 4:**
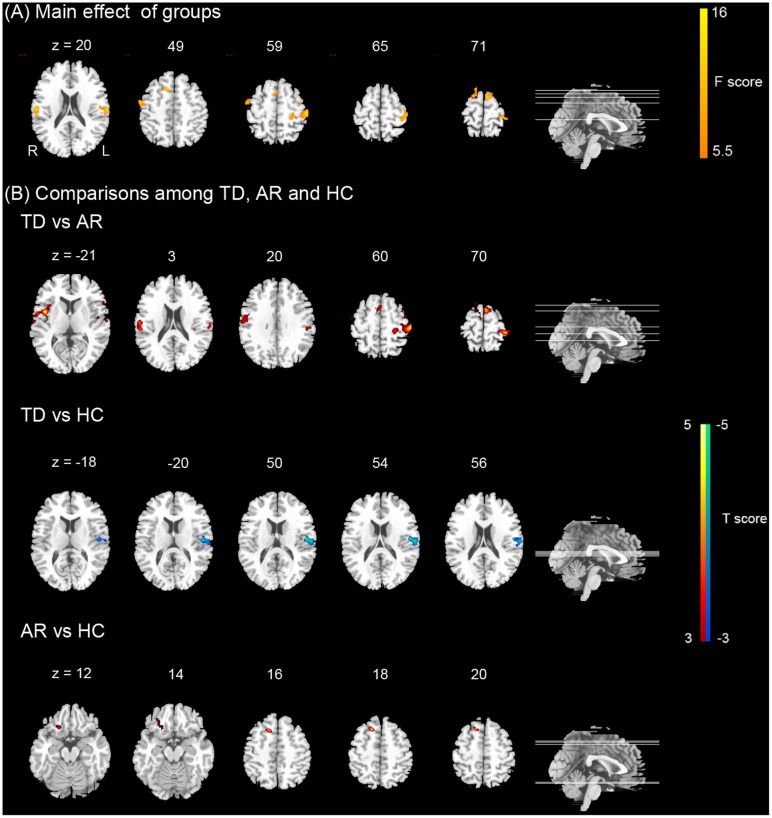
Results of the functional connectivity (FC) of the left cerebellum. **(A)** Shows the main effect of group on FC [one-way ANCOVA, *p* < 0.005 corrected for multiple comparison at the cluster level, AlphSim corrected, *F*(2,69)]. Color bar represents the *F*-value. **(B)** Show the differences on FC of the left cerebellum between TD, AR, and HC (*post hoc* two-sample *t*-test, *p* < 0.005 corrected for multiple comparison at the cluster level, AlphSim corrected). Color bar represents the *t*-value.

For the seed of the left cerebellum anterior lobe, TD patients showed significantly increased FCs in the left ventral and dorsal post-central gyrus, left superior frontal gyrus, right insula and orbital inferior frontal gyrus compared with AR patients (**Figure 4** and **Table 4**).

**Table 4:** The altered functional connections of seeds in the group level.

Seed	Region	MNI coordinates			Peak *F*-value	Peak *T*-value	Cluster size (mm^3^)
		*x*	*y*	*z*			
**Left cerebellum anterior lobe**	**Main effect of group**						
	R_ventral post-central gyrus	60	-15	15	9.17		1620
	L_ventral post-central gyrus	-63	-21	15	8.5		945
	R_superior frontal gyrus	15	21	51	9.3		513
	L_dorsal post-central gyrus	-30	-30	78	8.87		1269
	**TDs > ARs**						
	R_insula	42	0	3		4.6	945
	L_ventral post-central gyrus	-66	-21	18		3.52	1080
		-54	-12	27		3.33	621
		-57	-6	48		3.86	1674
	L_superior frontal gyrus	-6	9	69		4.4	1323
		0	15	54		3.98	1566
	L_dorsal post-central gyrus	-30	-33	78		4.51	4077
	R_orbit inferior frontal gyrus	23	18	-21		3.72	837
	**ARs > HCs**						
	L_ventral post-central gyrus	-60	-18	15		-4.11	1998
	**TDs > HCs**						
	R_orbit superior frontal gyrus	15	24	-24		3.83	648
	R_superior frontal gyrus	15	21	51		4.53	729
**Right superior frontal gyrus**	**Main effect of group**						
	L_posterior cingulate	-6	-39	9	9.95		918
	R_inferior parietal lobule	66	-39	24	8.08		513
	L_precuneus	-3	-57	48	7.55		702
	**TDs > HCs**						
	L_posterior cingulate	-6	-42	9		-3.97	540
	**ARs > HCs**						
	L_precuneus	-6	-57	48		-3.91	2349
	R_inferior parietal lobule	69	-36	21		4.07	810
**Left inferior frontal gyrus**	**Main effect of group**						
	L_cerebellum posterior lobe	-48	-66	-30	8.32		756
**Right middle frontal gyrus**	**Main effect of group**						
	L_cerebellum posterior lobe	-39	-066	-45	14.92		1728
	R_parahippocampa gyrus	27	-3	-27	6.9		486
	**TDs > HCs**						
	L_cerebellum posterior lobe	-39	-66	-48		-6.3	3078

*The results are altered functional connections of seeds among TDs, ARs, and HCs [one-way ANCOVA, *p* < 0.005 corrected for multiple comparison at the cluster level, AlphSim corrected, *F*(2,69)] and the comparisons between two groups (*post hoc* two-sample *t*-test, *p* < 0.005 corrected for multiple comparison at the cluster level, AlphSim corrected)*.

For the remaining seeds, no significant difference in FC was found between TD and AR patients (**Table 4**).

## Discussion

Our findings obtained using a combination of FCD and seed-based FC analyses provide consistent evidence for that the network disorganization of the brains in the two PD subtypes were different. These findings have important implications for understanding the neural substrates that underlie these disparate manifestations of PD.

These results indicate that AR and TD subgroups both represent altered network disorganization. The global FCD abnormalities between the three groups involved the premotor cortex, supplementary motor area (SMA), prefrontal region (e.g., middle frontal gyrus and superior frontal gyrus) and the cerebellum. The premotor cortex plays an important role in the temporal organization of sequential movements, the selection of movements and the generation of motor sequences from memory that fit into a precise plan (Deiber et al., 1991; Halsband et al., 1993). The SMA is critical for the planning and initiation of movements, particularly internally generated, self-paced movement sequences (Jenkins et al., 2000; Tanji and Hoshi, 2001). The prefrontal cortex plays an important role in the learning of new motor sequences (Jueptner et al., 1997). The cerebellum receives information from the motor cortex, and cerebellar output influences various neuronal populations in the motor cortex (Manto et al., 2012). Therefore, we postulated that the FCD of these sites differed between the PD group (AR and TD patients) and the HC group due to the importance of these sites for motor function.

Intriguingly, we found that there was increased global FCD in the frontal lobe in the AR patients compared with the TD patients. Previous studies have demonstrated that these related regions are associated with the AR subtype (Hanakawa et al., 1999). The SMA and the pre-SMA also play a crucial role in the pathogenesis of bradykinesia in PD (Cunnington et al., 2002; Esposito et al., 2013). The dysfunction of the SMA has been recognized as a crucial reason for akinesia (Zhang et al., 2016). Thus, we speculate that increased global FCD in the frontal lobe in AR patients may be the manifestation of an attempt to compensate for the weaker information processing function due to the pathogenesis of akinetic-rigidity even before the onset of gait symptoms. In addition, we found that global FCD in left middle temporal gyrus was increased in AR patients compared with the HC group and a significant negative correlation between age of disease onset and the FCD value in middle frontal gyrus of the AR patients. Several studies have confirmed that patients with late-onset PD have a greater tendency for early development of bradykinesia and rigidity (Wickremaratchi et al., 2011; Thenganatt and Jankovic, 2014), so this functional integration change may suggest that the AR subtype shows more aggressive symptom, manifested by earlier and more rapid physical decline (Thenganatt and Jankovic, 2014). What’s more, it is generally believed that the bilateral prefrontal cortices as well as middle temporal gyri are crucial nodes of the DMN (Raichle et al., 2001; Tessitore et al., 2012), and the AR subtype shows a more rapid development of cognitive decline (Karunanayaka et al., 2016). This result suggests that there is an early functional disruption of the DMN in AR prior to clinical evidence of cognitive impairment, and the AR patients were likely to invest more neural resources to compensate for the cognitive decline (Marras et al., 2002; Rajput et al., 2009).

We also found increased global FCD in the cerebellum of TD patients compared with the AR patients. Previous studies have shown that structural and functional changes in the cerebellum are highly related to tremor-related diseases, such as cortical myoclonic tremor (Buijink et al., 2013) essential tremor (Louis et al., 2006; Popa et al., 2011) and resting tremor in PD (Mure et al., 2011) these changes may also be related to the symptoms. Mounting evidence suggests that cerebellar glucose metabolism and blood flow are associated with the severity of tremor in PD (Fukuda et al., 2004; Benninger et al., 2009), especially the correlations of changes in tremor frequency within condition with rCBF in the contralateral cerebellum has been observed (Fukuda et al., 2004). Wang’s research showed that the enhanced Vim–cerebellum/dentate nucleus connectivity is associated with parkinsonian tremor; this connectivity is more strengthened as tremor becomes more severe (Wang et al., 2016). We also found that alterations in interhemispheric correlations in the cerebellum may be involved in the neuronal basis of the resting tremor symptoms of PD (Zhang et al., 2015b). Furthermore, we found a significant negative correlation between H-Y stage and the FCD value of the cerebellum anterior lobe in the PD group. We suspect that in TD patients, abnormal elevations of blood perfusion and greater mobilization of the cerebellum may constitute a potential compensatory mechanism for the cerebellar dysfunction of PD. One likely explanation is that this phenomenon presents a functional compensation for the defective basal ganglia (Wu and Hallett, 2005). Previous study showed that increased coupling between the subthalamic nucleus (STN) and cerebellum might underlie the neural substrate of PD tremors. Lower FC between the STN and putamen might underpin PD gait and posture disturbances, while higher FC between the STN and visual cortex might play a compensatory role (Zhang et al., 2016). But we find no significant difference in the basal ganglia and substantia nigra between two subtypes, just like our previously published study (Supplementary Data and Figure 2).

Previously, Zhang et al. (2015b) demonstrated abnormal FCD in PD and speculated that the observed FCD reductions in the ventral visual pathway might be responsible for the non-psychotic visual hallucinations in PD patients. They also speculated that higher FCD in the posterior cingulate cortex (PCC) and precuneus might be associated with the inability of PD patients to redirect attentional resources. To identify FC alteration related to PD motor symptoms and subtypes, we strictly controlled the inclusion criteria, and patients with visual hallucinations or cognitive dysfunction were carefully excluded. We found no altered FCD in the ventral visual pathway, PCC or precuneus. However, compared with controls, we did find increased global FCD in the fusiform gyrus in the AR patients. Because the fusiform gyrus is involved in visual information processing and recognition, such as face recognition and face hallucinations (Benninger et al., 2009; Blom, 2009) we speculate that non-psychotic visual hallucinations could affect FCD in PD earlier than we thought, even before the appearance of these symptoms.

We further investigated FC in the four altered FCD regions. FC analysis also showed altered FC between the frontal lobe and cerebellum in the TD patients compared with the AR patients, consistent with the notion that PD tremor is most likely related to combined impairment of the cerebello-cortical circuit. Considering these results together with the abnormal FCD that was observed in the two subtypes and the relationships with clinical data, we speculate that as part of the compensatory effects required for the generation of movements, more cerebellar resources are recruited in the TD subtype, whereas more frontal lobe resources are recruited in the AR subtype. Further research is needed to further explore the mechanisms underlying these changes.

We also found that the FCD provided good discrimination between the AR and TD patients. The diagnosis of different subtypes depended on UPDRS score (Jankovic et al., 1990; Stebbins et al., 2013) and we achieved relatively objective discrimination of the different subtypes, with good sensitivity (95%) and specificity (80%) for classifying PD patients. Previous studies have only attempted to use FC metrics to distinguish between PD and healthy individuals (Fernandez-Seara et al., 2015; Zhang et al., 2015a). The results demonstrate the importance of altered global FCD in discriminating the two subtypes. As we relied on an experienced neurologist for the diagnosis of the two subtypes in this study and as these findings have not been reported previously, the classification results presented here are preliminary, and further studies are required to corroborate our interpretation. The diagnostic power of FCD for PD subtypes also requires further investigation.

### Limitations

Some methodological limitations of this study should be mentioned. First, the small sample size and the relatively weak correction strategy employed (AlphaSim program) limited the conclusions that could be drawn; thus, a large number of participants should be included in future studies. Second, most of the PD patients were administered dopaminergic medications and a few with anticholinergics which might affect brain condition (Ehrt et al., 2010). Studies of drug-naïve individuals are warranted to exclude the effects of dopaminergic and anticholinergics medications on the findings. Longitudinal data are also required to further study the neuroimaging changes observed in the two subtypes. Third, the threshold (Tc) of the correlation coefficient used to define the functional connection was set at 0.6 in the FCD analysis based on prior knowledge, and this fixed value may lead to some false positive or negative findings. Flexible thresholds, such as a set of continuous thresholds, might be an alternative option for overcoming this limitation. We also evaluated the difference in FCD among groups using five thresholds (from 0.3 to 0.7 at intervals of 0.1), and similar findings were observed across several calculations using different thresholds (Supplementary Data and Figure 1). Lastly, considering other literature on PD pathophysiology, there are certainly other ways to subtype PD patients, i.e., according to different genetic backgrounds and/or clinical presentations. Our study, however, focused on TD versus AR subtype, one of the first clinical sub-types described in Jankovic et al. (1990). Future studies, therefore, are warranted to understand the relevance of resting state FC differences between PD subtypes other than TD versus AR.

## Author Contributions

XH: project execution; MRI data acquisition; healthy control recruitment and demographic information acquisition; writing of manuscript. YJ: MRI data analysis, statistical analysis; writing of manuscript; revision of this manuscript. XJ: MRI data analysis. JZ: manuscript review and revision. ML: MRI data acquisition. JL: participant recruitment and clinical data acquisition. YZ: participant recruitment and clinical data acquisition. DY: manuscript review and revision. CL: project conception, manuscript review and critique. JW: project conception, manuscript review and critique.

## Conflict of Interest Statement

The authors declare that the research was conducted in the absence of any commercial or financial relationships that could be construed as a potential conflict of interest.

## Footnotes

1 http://www.neuro.uestc.edu.cn/NIT.html

2 https://afni.nimh.nih.gov/afni/doc/manual/AlphaSim.pdf
