# Supplementary material for: Altered Functional Connectivity Density in Subtypes of Parkinson’s Disease
Source: Front Hum Neurosci. 2017 Sep 20;11:458. doi: 10.3389/fnhum.2017.00458 (PMC5609108; doi:10.3389/fnhum.2017.00458)
Supplement: Supplementary file 1 [file Presentation_1.PDF]

Supplementary Material

Altered Functional Connectivity Density in Subtypes of Parkinson's Disease

Xiaofei Hu1,†, Yuchao Jiang2,† Xiaomei Jiang[3], Jiuquan Zhang[1], Minglong Liang[1], Jing Li[4], Yanling Zhang, [4] Dezhong Yao,[2] Cheng Luo[2,*], Jian Wang[1,*]

[1] Department of Radiology, Southwest Hospital, Third Military Medical University, Chongqing, 400038, P.R. China

[2] Key Laboratory for NeuroInformation of Ministry of Education, School of Life Science and Technology, University of Electronic Science and Technology of China (UESTC), Chengdu, 610054, P.R. China

[3]Department of Centre for Disease Prevention and Control, Chengdu Military Region, Chengdu, 610021, P.R. China

[4]Department of Neurology, Southwest Hospital, Third Military Medical University, Chongqing, 400038, P.R. China

†Co-first Author

*** Correspondence Address:**

Jian Wang, Department of Radiology, Southwest Hospital, Third Military Medical University, Chongqing, 400038, P.R. China Tel: +86-23-6876-5419, Fax: +86-23-6546-3026, E-mail: wangjian_811@yahoo.com

Cheng Luo, Key Laboratory for NeuroInformation of Ministry of Education, School of Life Science and Technology, University of Electronic Science and Technology of China (UESTC), Chengdu, 610054, P.R. China E-mail: chengluo@uestc.edu.cn

1 Supplementary Data

Based on prior knowledge, the threshold was set at 0.6 in the FCD analysis. To avoid bias in the findings potentially introduced by the choice of threshold, we calculated the FCD using a series of thresholds (from 0.3 to 0.7 in intervals of 0.1). Fortunately, the differences in FCD value among three groups remained similar across several thresholds (0.3-0.7). The results are shown in Supplementary Figure 1. We did not include 0.8 as the FCD threshold because this high threshold may lead to lower sensitivity due to the reduced dynamic range of the FCD value(Tomasi and Volkow, 2010). Therefore, we have reported the results using the 0.6 threshold in the manuscript.

Several concerns about censoring have been raised in various studies(Power et al., 2015). One concern relates to degrees of freedom. Censoring motion-contaminated data leads to fewer degrees of freedom in subjects who move more. This could result in biases that covary with factors of interest, such as less data in higher-moving clinical cohorts. Another concern is that correlation estimates may become excessively noisy if data is removed. Furthermore, the distribution of correlations will widen and is more likely to contain "extreme" values after censoring. Thus, we did not apply volume censoring because of these concerns. In addition, there is a standardized MRI data quality management process employed in our research, and after scanning, the MRI data were inspected to exclude subjects with gross abnormalities in the brain and those who did not meet the head-motion criterion. Therefore, all of the data in the study were of high quality, and no subject was excluded according to the head-motion criterion. In addition, there is no differences among three groups in term of FD (F=1.302, p=0.278, ANOVA) (Supplementary Table 1).

We also performed a functional connectivity analysis with basal ganglia as seeds since the specific defect of the PD patients. The bilateral putamen and caudate were chosen as the seeds separately according previous studies(Eggers et al., 2012; Zhang et al., 2015b). The ANCOVA was used to determine the group differences among TD, AR and HC. The regions with significant group differences were extracted and then the LSD post-hoc analyses were performed to compare the differences between any two groups.

Result

ANCOVA showed the significant group differences for the functional connectivity between the right putamen and orbitofrontal cortex(OFC), the left putamen and cerebellum, as well as right caudate and postcentral gyrus (Supplementary Figure 2). Post-hoc analyses showed that for these functional connections, both TD and AR exhibited significant differences with HC. In addition, for the functional connectivity of right putamen and orbitofrontal cortex, the AR was different from the TD.

Discussion

The altered functional connectivity between basal ganglia and cerebellum, and between basal ganglia and frontal cortex (orbitofrontal cortex and postcentral gyrus) highlight the involvement of cerebello-thalamo-cortical (CTC) circuit both in AR and TD subtypes, and underscore the importance of integrating mesolimbic-striatal and CTC loops in understanding neural systems of akinesia and rigidity, as well as resting tremor in PD, which is consistent with our previous study(Zhang et al., 2015a).

FC analysis also showed altered FC between the putamen and orbitofrontal cortex in the AR patients compared with the TD patients. The orbitofrontal cortex plays an important role in in the cognitive processing of decision-making(Kringelbach, 2005) and the AR subtype shows a more rapid

development of cognitive decline(Karunanayaka et al., 2016). This result proved again that there is a functional disruption involved in cognitive impairment in AR group.

2 Supplementary Figures and Tables

2.1 Supplementary Figures

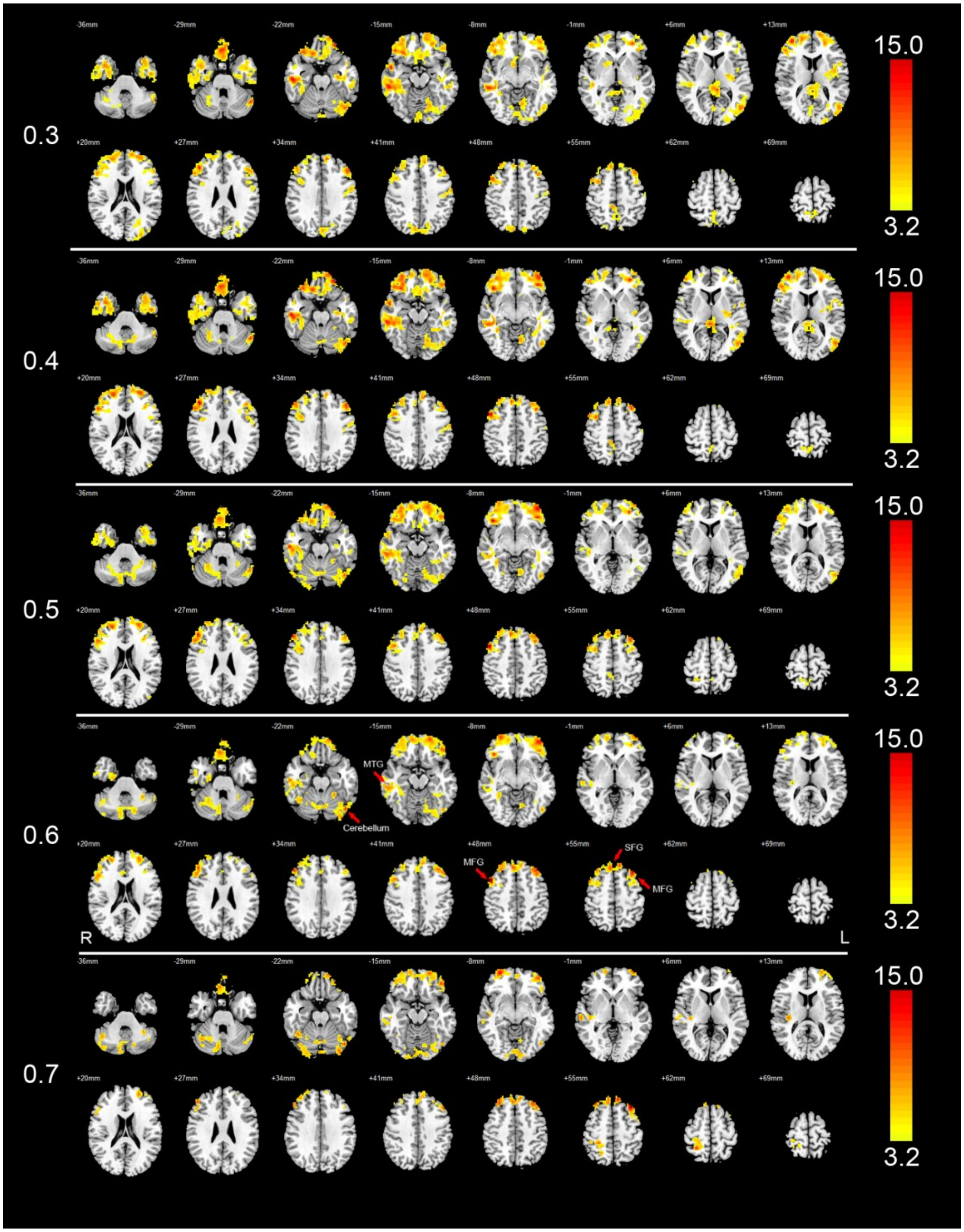

Supplementary Figure 1. Results of the FCD threshold analyses. Figure 1 shows the spatial distribution of the average global functional connectivity density (FCD) for TD, AR and HC using a series of thresholds (0.3-0.7) (P<0.005, Alphasim corrected). Color bar represents the F-value.

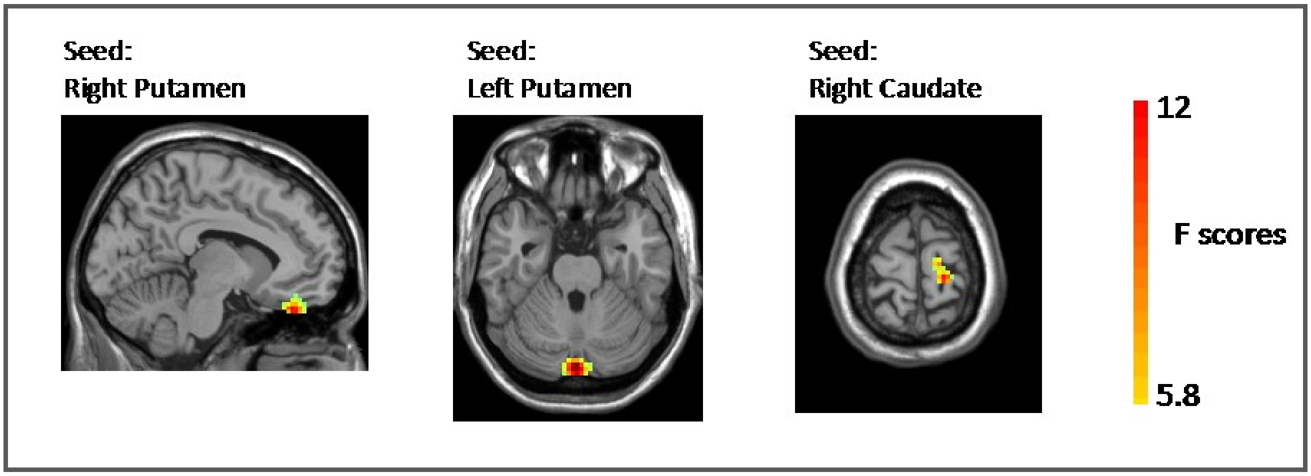

Supplementary Figure 2. Results of the FC analyses with basal ganglia as seeds. Figure shows the main effect of group on FC (one-way ANCOVA, P<0.005 corrected for multiple comparison at the cluster level, AlphSim corrected, F(2, 69)). Color bar represents the F-value.

2.2 Supplementary Tables

Table 1. The difference among three groups in term of FD.

Group	Mean FD	STD of FD
TD	0.0852	0.0612
AR	0.0980	0.1049
HC	0.0655	0.0348

STD: standard deviation

Eggers, C., Pedrosa, D.J., Kahraman, D., Maier, F., Lewis, C.J., Fink, G.R., et al. (2012). Parkinson subtypes progress differently in clinical course and imaging pattern. *PLoS One* 7(10)**,** e46813. doi: 10.1371/journal.pone.0046813.

Karunanayaka, P.R., Lee, E.-Y., Lewis, M.M., Sen, S., Eslinger, P.J., Yang, Q.X., et al. (2016). Default mode network differences between rigidity-and tremor-predominant Parkinson's disease. *Cortex* 81**,** 239-250.

Kringelbach, M.L. (2005). The human orbitofrontal cortex: linking reward to hedonic experience. *Nat Rev Neurosci* 6(9)**,** 691-702. doi: 10.1038/nrn1747.

Power, J.D., Schlaggar, B.L., and Petersen, S.E. (2015). Recent progress and outstanding issues in motion correction in resting state fMRI. *Neuroimage* 105**,** 536-551. doi: 10.1016/j.neuroimage.2014.10.044.

Tomasi, D., and Volkow, N.D. (2010). Functional connectivity density mapping. *Proc Natl Acad Sci U S A* 107(21)**,** 9885-9890. doi: 10.1073/pnas.1001414107.

Zhang, J., Wei, L., Hu, X., Xie, B., Zhang, Y., Wu, G.-R., et al. (2015a). Akinetic-rigid and tremor-dominant Parkinson's disease patients show different patterns of intrinsic brain activity. *Parkinsonism Relat Disord* 21(1)**,** 23-30.

Zhang, J., Wei, L., Hu, X., Xie, B., Zhang, Y., Wu, G.R., et al. (2015b). Akinetic-rigid and tremor-dominant Parkinson's disease patients show different patterns of intrinsic brain activity. *Parkinsonism & Related Disorders* 21(1)**,** 23-30.
